# Genome-Wide Insights into Intermittent Milking Behavior of Pandharpuri Buffalo

**DOI:** 10.3390/cimb48010101

**Published:** 2026-01-19

**Authors:** Akshata Patil, Parth Gaur, Pritam Pal, Rani Alex, Supriya Chhotaray, Ravi Kumar Gandham, Vikas Vohra

**Affiliations:** 1 Division of Animal Genetics and Breeding, ICAR-National Dairy Research Institute, Karnal 132001, Haryana, India; akshatapatil12645@gmail.com (A.P.); parthgaur@gmail.com (P.G.); pritamvet1@gmail.com (P.P.); ranialex01vet@gmail.com (R.A.); 2 Animal Genetics and Breeding, ICAR-Central Institute for Research on Buffalo, Hisar 125001, Haryana, India; supriya.chhotaray@gmail.com; 3 ICAR-National Bureau of Animal Genetic Resources, Karnal 132001, Haryana, India; gandham71@gmail.com

**Keywords:** Pandharpuri buffalo, intermittent milking, selection signatures, DCMS approach

## Abstract

Buffaloes (*Bubalus bubalis*) are central to the dairy and agricultural economy, contributing high-quality milk, meat, draft power, and manure. Rich milk composition, the ability to utilize low-quality roughage, and strong disease resistance make buffaloes indispensable across diverse production systems. Among India’s major dairy breeds—Murrah, Nili-Ravi, Jaffarabadi, Surti, Bhadawari, Mehsana, and Nagpuri, none exhibit the distinctive trait of intermittent milking, which is uniquely observed in the Pandharpuri buffalo, a registered indigenous breed of Maharashtra. Despite coexisting with dominant dairy breeds such as Murrah, Pandharpuri buffalo is considered to possess primitive riverine ancestry and may represent one of the ancestral lineages from which several Indian breeds evolved. Its evolutionary relevance and unique intermittent milking capacity underscore the need to understand its genomic architecture. To address this, we applied whole-genome resequencing and the De-Correlated Composite of Multiple Signals (DCMS) approach to identify within-breed selection signatures. Our analyses identified 1337 candidate genes, including several linked to milk production, particularly those relevant to the physiological capacity for intermittent milking. Notable genes included *ERBB4*, *ESR1*, *SYK*, *INSR*, *PTPN11*, *VAV3*, *MAPK3*, and *PRKG1*. These signatures provide insights into genomic regions and biological pathways that may be involved in lactation-related processes relevant to intermittent milking. The identified genomic regions offer promising targets for functional validation and future genome-informed breeding strategies aimed at conserving this unique indigenous germplasm while improving lactation efficiency and resilience.

## 1. Introduction

The advent of Next-Generation Sequencing (NGS) technologies, particularly whole-genome sequencing (WGS), has transformed livestock genetics. With hundreds of thousands of genomes now sequenced worldwide, WGS has become the predominant tool for genomic analysis [1]. By enabling the identification of genome-wide variation, WGS has unraveled the genetic architecture of complex traits and facilitated the discovery of candidate genes and molecular markers for livestock improvement [2,3]. Beyond these applications, WGS data can be exploited to detect genomic footprints shaped by evolutionary forces such as genetic drift and selection, with genome-wide scans enabling the identification of regions influenced by both natural and artificial pressures [4,5,6]. Positive selection drives the increase in frequency of advantageous alleles, leaving distinct genomic signatures that deviate from neutral expectations [7,8,9,10]. Detecting these signatures helps pinpoint genomic regions that have undergone past or ongoing selection, many of which harbor loci linked to economically important and adaptive traits. Such candidate regions provide valuable targets that can be further explored and incorporated into livestock genetic improvement strategies [11].

In livestock species, genome-wide selection signature analyses have revealed candidate genes for production traits, disease resistance, and environmental adaptation, while also uncovering breed-specific adaptive mechanisms, particularly in indigenous and locally adapted populations [12,13,14,15,16,17,18]. Beyond trait-focused applications, such analyses enhance genetic characterization of livestock populations, support the conservation of native germplasm, and provide a framework for understanding the evolutionary forces shaping phenotypic diversity [6]. Several statistical approaches have been developed to detect selection signatures, including frequency spectrum–based tests such as Tajima’s D [19], nucleotide diversity (π) [20], Fay and Wu’s H statistic [21], and the composite likelihood ratio test (CLR) [22], as well as haplotype-based statistics such as integrated Haplotype Score (iHS) [23], and number of Segregating Sites by Length (nSL) [24]. While informative, individual statistics capture only specific aspects of the selection process, and reliance on a single method may reduce sensitivity to weak or complex selection signals, resulting in inconsistent detection across the genome [25]. To overcome these limitations, composite approaches integrating multiple complementary statistics have been developed. Among these, the De-Correlated Composite of Multiple Signals (DCMS) framework accounts for correlations among individual tests, thereby improving detection power and positional accuracy [26]. The effectiveness of DCMS has been demonstrated in several livestock studies, where it successfully identified biologically meaningful selection signals associated with adaptation and economically important traits in livestock species [27,28,29,30,31,32,33]. Such integrative approaches are particularly valuable for small, localized, and indigenous breeds that harbor unique functional variation and distinct genomic diversity [34].

**Figure 1:**
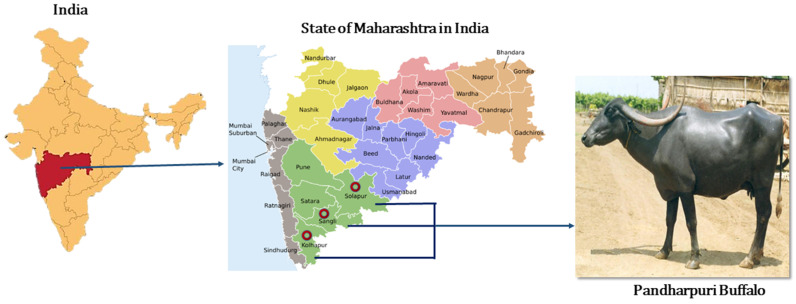
Home tract of the registered Indian breed Pandharpuri buffalo *(Bubalus bubalis*) in Kolhapur, Solapur, and Sangli districts of Maharashtra, India.

The Pandharpuri buffalo, a registered breed native to Maharashtra, India, represents a distinctive and valuable component of Indian germplasm (Figure 1). Characterized by its sword-shaped horns and a remarkable ability to tolerate intermittent milking, this breed plays an important role in rural dairy systems and retains traits of biological and evolutionary importance. In traditional practice, farmers often carry the animal from house to house, supplying milk directly to customers; the buffalo is milked multiple times throughout the route according to demand, and despite these irregular and repeated milking intervals, it continues to produce milk efficiently. This unique behavioural adaptation is well-documented in the breed descriptor and forms a defining feature of the Pandharpuri buffalo. This milking adaptability may be supported by physiological mechanisms unique to buffaloes. One such example is Doka, a phenomenon involving transient teat engorgement during estrus, commonly used by farmers as an indicator of reproductive status [34]. This engorgement results from PGF2α-induced oxytocin release even in the absence of external stimulation [35], reflecting a tightly coordinated reproductive–mammary hormonal axis. While not directly related to milking behaviour, this heightened endocrine responsiveness suggests a mammary system capable of rapid physiological adjustment.

From an evolutionary perspective, these physiological features align with the domestication history of river buffaloes in the Indian subcontinent. Historical and archaeological evidence indicates that regions including the Mehsana, Surti, and Pandharpuri tracts were important centers for domestication and diversification of riverine buffaloes [36]. Accordingly, the Pandharpuri breed likely represents a primitive population that contributed to the genetic foundation of other Indian breeds. Despite its evolutionary relevance and distinctive milking phenotype, the genomic basis underlying intermittent milking in Pandharpuri buffalo remains largely unexplored. Considering its intermittent milking pattern, evolutionary significance, localized adaptation, and genetic distinctiveness, the present study applied whole-genome resequencing and the DCMS framework to detect within-breed selection signatures in Pandharpuri buffalo. To the best of our knowledge, this is the first investigation to employ a DCMS-based genome-wide selection scan in this breed. By integrating multiple complementary selection statistics, this approach enables the identification of genomic regions and candidate genes potentially involved in adaptive processes and breed-specific characteristics, including traits related to intermittent milking. These findings provide insights into the genetic architecture of this unique germplasm and offer a foundation for future functional validation, conservation strategies, and genome-informed breeding programs.

## 2. Materials and Methods

### 2.1. Whole-Genome Resequencing and Variant Detection

The dataset comprised 15 Pandharpuri buffaloes, including four newly sequenced individuals and eleven publicly available whole-genome datasets reported by Dutta et al. (2020) [37]. The newly sequenced animals were four adult buffaloes sampled from four different herds belonging to distinct owners across villages within the Solapur district of Maharashtra, India, which lies centrally within the recognized Pandharpuri breeding tract encompassing Kolhapur, Solapur, and Sangli districts. Animals were selected based on established breed descriptors, and farmer-reported pedigree and ownership records were used to minimize the inclusion of closely related individuals. In addition, potential cryptic relatedness was assessed using genome-wide identity-by-descent (IBD) estimates, and no evidence of close relationships was detected among the sampled individuals. The eleven previously published genomes were derived from Pandharpuri buffaloes sampled within the recognized native breeding tract of Maharashtra, as reported in the original study [37]. Although fine-scale village-level metadata were not available for these publicly archived genomes, they represent the same indigenous population.

Genomic DNA was extracted using the phenol–chloroform protocol [38], and quality-checked prior to sequencing on the Illumina HiSeq platform (150 bp paired-end; ~44.5× coverage). Raw reads were subjected to quality control (FastQC; [39]) and adapter/quality trimming (Fastp; [40]). High-quality reads were aligned to the Murrah reference genome (GCF_019923935.1) using BWA-MEM [41]), with indexing [42] and dictionary creation performed via PicardTools. Post-alignment processing included sorting, duplicate removal, and read group assignment. Single Nucleotide Polymorphisms (SNPs) were identified with GATK HaplotypeCaller [43] followed by joint genotyping across all samples. These SNPs were used for downstream analyses.

### 2.2. High-Confidence Variant Selection and Phasing

Variant filtering retained only high-confidence, biallelic SNPs. Filtering was implemented with BCFtools v1.10.2 (minimum read depth ≥ 10; maximum depth 500; QUAL ≥ 30), and additional hard filters applied in GATK VariantFiltration v4.6.0.0 excluded SNPs with QD < 2.0, FS > 60.0, SOR > 4.0, MQ < 40.0, MQRankSum < –12.5, or ReadPosRankSum < –8.0. For downstream selection signature analyses, only autosomal SNPs were retained. Additional filtering was conducted in PLINK v1.9 [44] using thresholds of sample call rate > 0.9, SNP call rate > 0.9, and Minor Allele Frequency (MAF) > 0.05. For haplotype-based methods, iHS and nSL, the chromosome-wise SNP datasets were phased with SHAPEIT v2.17 [45].

### 2.3. Computation of Intra-Population Selection Statistics

To identify genomic regions under selection in Pandharpuri buffalo, we applied the DCMS framework, which integrates multiple statistics into a single composite score. Four intra-population statistics—Tajima’s D [19], nucleotide diversity (π) [20], iHS [23], and nSL [24] were used. Tajima’s D and π were estimated in non-overlapping 50 kb windows using VCFtoolsv0.1.16 [46]. VCFtools was selected because it provides efficient and validated implementations of site frequency spectrum–based summary statistics directly from Variant Call Format (VCF) files, the standard output of whole-genome resequencing pipelines. Its ability to handle large-scale datasets, support window-based analyses, and its widespread adoption in livestock genomic diversity and selection signature studies make it a robust and appropriate tool for population genomic analyses [15,28,31].

The iHS and nSL statistics were computed from phased SNP data using selscan v1.3.0 with the—ihs and—nsl commands, respectively and normalized per chromosome using the—norm option in selscan [47]. Although Tajima’s D and nucleotide diversity (π) were estimated using non-overlapping 50 kb windows, their values were assigned to all SNPs falling within the corresponding window to enable SNP-level integration within the DCMS framework. In contrast, iHS and nSL were directly computed at the SNP level from phased haplotypes. This approach ensured that all four selection statistics were represented at the SNP level for DCMS calculation, consistent with previous DCMS-based selection signature studies.

### 2.4. Composite Selection Signature Analysis Using DCMS

To detect genomic regions under positive selection, we applied the DCMS approach [26] using four statistics: iHS, nSL, Tajima’s D, and nucleotide diversity (π). A SNP-level input file was processed in R v4.1.1 with the MINOTAUR, rrcovNA, MASS, data.table, and qvalue packages. Genome-wide *p*-values for each statistic were derived using stat_to_pvalue () (MINOTAUR) with one-tailed tests (right-tailed for iHS and nSL; left-tailed for Tajima’s D and π). A robust correlation matrix among statistics was then estimated using CovNAMcd() (rrcovNA) with the Minimum Covariance Determinant method (α = 0.75, nsamp = 50,000). This matrix was supplied to the DCMS () function (MINOTAUR) to calculate composite DCMS values for each SNP. The DCMS statistics is computed at any given loci *l* as follows:

(1) $${DCMS}_{l}=\;\sum_{t=1}^{n}\frac{log(\frac{1-p_{lt}}{p_{lt}})}{\sum_{i=1}^{n}\left|r_{it}\right|}$$

where, *p_lt_* represents the *p*-value of the *t*th statistic at the *l*th genomic position, while *r_it_* corresponds to the weight assigned to each statistic, derived from the genome-wide correlation coefficients among pairs of univariate tests. Composite DCMS values for each SNP were fitted to a normal distribution using the rlm () function (MASS package, v7.3-65), and the estimated mean (μ) and standard deviation (σ) were used to derive right-tailed *p*-values with the pnorm () function.

To identify genomic regions under selection, DCMS scores were converted into SNP-level *p*-values by fitting a normal distribution using a robust linear model, followed by one-sided (right-tailed) testing. Multiple testing correction was applied using the False Discovery Rate (FDR) procedure implemented in the R using qvalue package. SNPs with *q*-values < 0.05 were considered statistically significant, resulting in 26,814 significant SNPs across the autosomal genome. Significant SNPs were then consolidated into putative selection sweep regions using a 50 kb genomic binning strategy. Specifically, consecutive significant SNPs (*q* < 0.05) located within the same or adjacent 50 kb intervals were grouped to define core candidate regions. To account for local linkage disequilibrium (LD) and prevent fragmentation of biologically linked signals, each core region was extended bi-directionally until flanked by SNPs with *q*-values > 0.10, methodological strategy broadly consistent with approaches adopted in previous DCMS-based and genome-wide selection signature studies [6,15,28]. Additionally, adjacent extended regions were merged if the intervening distance was ≤50 kb, ensuring continuous sweep regions reflective of LD blocks. This multi-tiered approach led to the identification of 1405 candidate selection sweep regions across the genome.

DCMS-derived *p*- and *q*-values were used to rank SNPs genome-wide, and the resulting 1405 selection sweep regions were converted into BED format and intersected with the Bubalus bubalis reference annotation (GTF) using BEDTools [48] to identify candidate genes within selected regions.

### 2.5. Functional Annotation and Hub Gene Identification

Candidate genes detected within selection sweep regions were subjected to functional enrichment analysis using DAVID v6.8 [49] to provide insights into their biological roles and overrepresented KEGG pathways; enrichment results are reported using nominal *p*-values from Fisher’s exact test. The background gene set used for DAVID enrichment consisted of annotated Bubalus bubalis genes. Gene–gene interaction networks were then generated through the STRING database [50], and the resulting networks were imported into Cytoscape v3.9.0 [51]. The CytoHubba plugin [52] was employed to prioritize and identify hub genes within these networks.

## 3. Results

Whole-genome resequencing of 15 Pandharpuri buffaloes generated a total of 13.89 billion raw reads, of which 9.11 billion high-quality reads passed quality control and 9.01 billion were successfully aligned to the Murrah reference genome, achieving an average mapping rate of ~98.9%. Across the 15 Pandharpuri buffalo genomes analyzed, sequencing coverage varied according to data source. Among the publicly available genomes, four individuals were sequenced at an average coverage of ~8×, while the remaining eleven genomes (including all newly sequenced individuals) exhibited higher coverage ranging from ~28× to ~45× (Supplementary Table S1). Overall, the dataset was dominated by high coverage genomes, ensuring reliable variant detection for within-breed selection signature analyses. The mean mapping quality across samples was 56.68, confirming the reliability of alignments. Initial variant calling identified 27.0 million SNPs. Following rigorous quality control, including depth-based filtering, hard filtering with GATK, and subsequent filtering in PLINK based on sample call rate greater than 0.9, SNP call rate greater than 0.9, and MAF greater than 0.05, a final dataset of 20,492,591 high-confidence biallelic SNPs was retained for downstream selection signature analysis.

### 3.1. Genome-Wide Detection of Putative Selection Signatures Using DCMS

**Figure 2:**
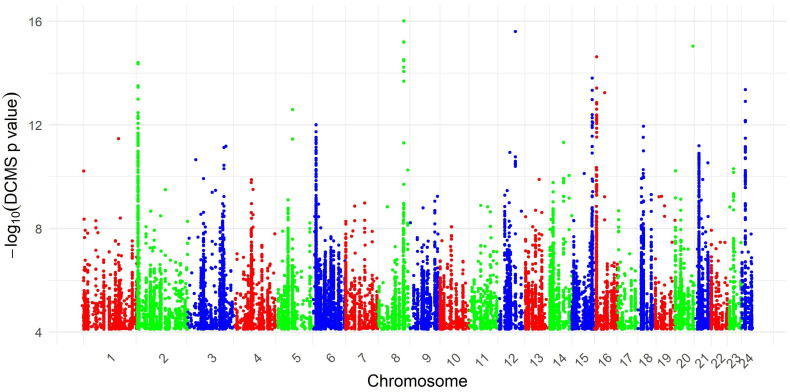
Manhattan plot displaying the distribution of SNPs with significant DCMS *q*-values (*q* < 0.05) across bovine autosomes. Each point represents a SNP, color-coded by chromosome, with the *y*-axis indicating the negative log-transformed DCMS *q*-value (−log10).

**Figure 3:**
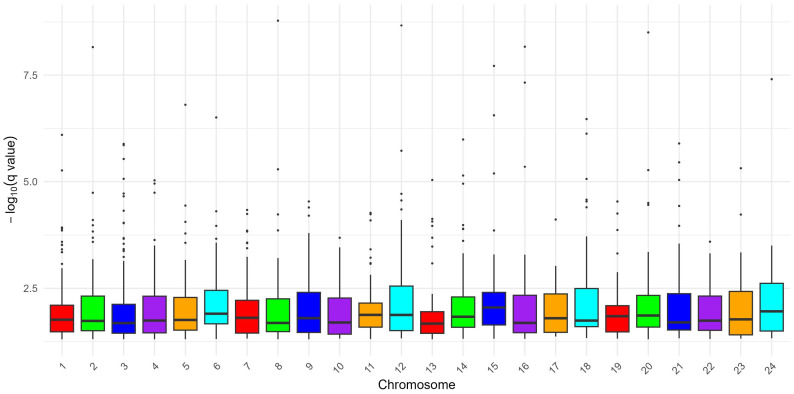
Distribution of *q*-Values Across Chromosomes in Pandharpuri Buffalo. (Boxplot displaying the distribution of q-values from the DCMS analysis across chromosomes (1–24) in Pandharpuri buffalo, with significant thresholds (*q* < 0.05) indicated. The *y*-axis represents −log10 (*q*-value), and boxes depict the interquartile range and whiskers showing variability).

A genome-wide scan employing the DCMS method identified 26,814 SNPs exhibiting significant empirical *q*-values (*q* < 0.05), indicative of putative selection signatures in the Pandharpuri buffalo population (Figure 2). Integration of DCMS statistics delineated 1405 discrete genomic regions under selection, with a mean length of 25.63 kb (approximately 0.0256 Mb) and a cumulative span of 36.01 Mb across the autosomes. The Manhattan plot depicting the chromosomal distribution of these selection signals is illustrated in Figure 3, revealing heterogeneous clustering with pronounced peaks on multiple chromosomes. Examination of the genome-wide distribution of DCMS scores indicated appropriate calibration, with no evidence of pronounced inflation or deflation, supporting the robustness of the DCMS-based selection signals. The strongest selection signal was detected on chromosome 8 (CHR 8: 93.75–93.95 Mb), encompassing 131 annotated genes distributed across adjacent intervals. Additional prominent regions included chromosome 12 (66.04–66.10 Mb), chromosome 20 (64.40–64.45 Mb), and chromosome 16 (8.73–8.99 Mb), as detailed in Table 1.

The 1405 putative selective sweep regions harboured 1337 unique genes, derived from the within-population DCMS distribution. The full list of 1405 DCMS-based selection sweep regions is provided in Supplementary Table S2. The top 20 genomic regions showing the strongest selection signals in Pandharpuri buffalo are summarized in Table 1. Notable candidate genes encompassed *PTPN11* (protein tyrosine phosphatase non-receptor type 11), *INSR* (insulin receptor), *SYK* (spleen-associated tyrosine kinase), *ERBB4* (erb-b2 receptor tyrosine kinase 4), *ESR1* (estrogen receptor 1), *VAV3* (vav guanine nucleotide exchange factor 3), *MAPK3* (mitogen-activated protein kinase 3), *PRKG1* (cGMP-dependent protein kinase).

### 3.2. Biological Processes and Pathways Enriched Among Candidate Genes

DAVID-based functional enrichment analysis of the 1337 candidate genes yielded 58 enriched terms (Fisher’s exact test, *p* < 0.05), comprising 17 biological processes (BP), 7 cellular components (CC), 16 molecular functions (MF), and 18 Kyoto Encyclopedia of Genes and Genomes (KEGG) pathways (Supplementary Table S3). Prominent GO terms included intracellular signal transduction (GO:0035556; 23 genes, fold enrichment = 2.00), calcium ion transmembrane transport (GO:0070588; 7 genes, fold enrichment = 3.50), endoplasmic reticulum tubular network organization (GO:0071786; 3 genes, fold enrichment = 9.22), cell surface (GO:0009986; 12 genes, fold enrichment = 2.10), calcium ion binding (GO:0005509; 46 genes, fold enrichment = 1.67), and ATP binding (GO:0005524; 78 genes, fold enrichment = 1.41), T cell receptor signaling pathway (GO:0050852, 5 genes, 3.84). These annotations underscore cellular mechanisms pivotal for metabolic homeostasis and stress resilience.

KEGG pathway enrichment highlighted significant overrepresentation in the Wnt signaling pathway (bbub04310; 20 genes, fold enrichment = 2.47), cAMP signaling pathway (bbub04024; 22 genes, fold enrichment = 1.91), insulin secretion (bbub04911; 10 genes, fold enrichment = 2.49), GnRH signaling pathway (bbub04912; 10 genes, fold enrichment = 2.25), and aldosterone synthesis and secretion (bbub04925; 10 genes, fold enrichment = 2.16). Collectively, these pathways implicate the identified regions in regulatory cascades governing milk production (e.g., via insulin and cAMP-mediated glucose uptake) and adaptation (e.g., via aldosterone for electrolyte balance under heat stress), mirroring observations in temperate and tropical dairy breeds [15,28].

### 3.3. Protein-Protein Interaction Network Analysis and Identification of Key Hub Genes

**Figure 4:**
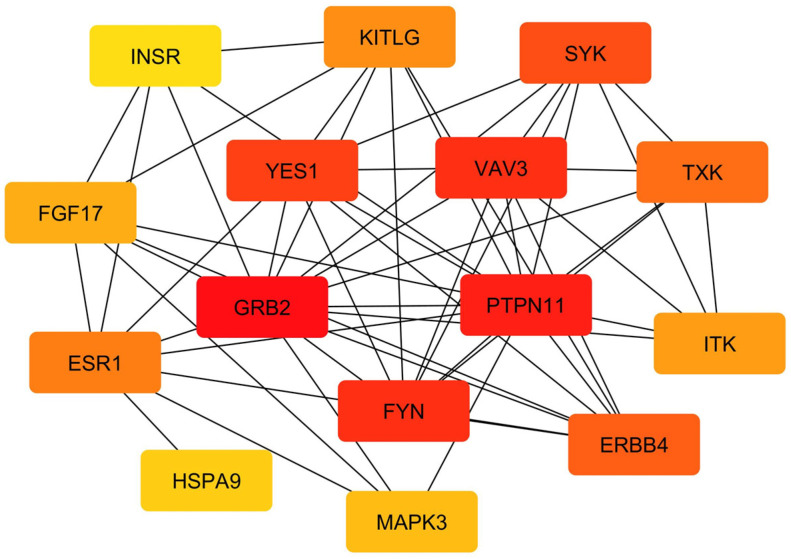
Protein–protein interaction (PPI) network analysis of the top 15 hub genes identified among candidate selection regions. Node color intensity reflects connectivity, with red indicating highest degree centrality.

To elucidate functional interconnectivity among the candidate proteins, a protein-protein interaction (PPI) network was constructed using the STRING database (v12.0) at a high-confidence threshold (interaction score > 0.70). Of the 1337 input genes, 856 were successfully mapped, forming a network with 856 nodes and 360 edges. The average node degree was 0.84, with the observed interactions exceeding random expectations (PPI enrichment *p*-value = 1.61 × 10^−10^), suggesting biologically cohesive modules. Hub genes were prioritized using the Maximum Clique Centrality (MCC) algorithm implemented in CytoHubba (Cytoscape v3.10.2), revealing key nodes potentially driving network topology and trait associations (Figure 4). The PPI analysis was used primarily to support and prioritize candidate genes consistently identified across DCMS-based selection scans and functional enrichment analyses, rather than as an independent source of biological inference. The top 15 hub genes identified from the STRING protein–protein interaction network, ranked using the Maximum Clique Centrality (MCC) method, are provided in Supplementary Table S4.

## 4. Discussion

Understanding the genetic mechanisms underlying breed-specific adaptation requires moving beyond phenotypic observation to unravel the genomic architecture shaped by natural and artificial selection. Livestock breeds maintained under diverse ecological and management pressures, such as thermal stress, feed scarcity, or irregular milking, accumulate unique combinations of alleles that confer survival and productivity advantages. Uncovering such adaptive signatures necessitates a genome-wide approach capable of detecting both common and rare variants across the entire spectrum of genetic diversity. WGS provides the most comprehensive platform for variant discovery, enabling the identification of multiple forms of genetic variation, including SNPs, insertions and deletions (InDels), structural variants (SVs), and copy number variations (CNVs) [53]. Unlike SNP arrays or reduced-representation approaches such as ddRAD-seq, WGS offers superior resolution by capturing rare, low-frequency, and breed-specific variants that are often missed by conventional genotyping platforms. This ability to detect the full spectrum of genomic variation is particularly valuable for indigenous breeds, where adaptive alleles underlying environmental resilience and unique production traits frequently reside in less-characterized regions of the genome.

The Pandharpuri buffalo, is traditionally managed under intermittent milking schedules, which reflect a combination of management practices and intrinsic lactation biology. Such milking patterns have direct implications for milk yield, mammary gland physiology, and metabolic homeostasis, highlighting adaptive strategies that allow this breed to maintain lactation despite irregular milking intervals. Ruminant species exhibit diverse lactation strategies; for example, the ATM (“Anytime Milk”) goat is renowned for nearly continuous milk production, demonstrating a genetic predisposition for sustained lactation. While not directly comparable, these differences illustrate the spectrum of lactation adaptability among domesticated ruminants and underscore the importance of breed-specific physiological and genetic mechanisms in shaping milking responses. Considering the significance of this unique breed and the advantages of high-resolution genomic analysis, the present study employed whole-genome resequencing of 15 Pandharpuri buffaloes to identify genome-wide signatures of selection that may have arisen through generations of adaptation. The majority of genomes exhibited coverage exceeding 30×, which is particularly advantageous for studies involving indigenous breeds with naturally small breeding populations and limited sample sizes, as it enhances variant detection accuracy, minimizes false negatives, and enables robust population-level genomic inference [54].

Selection signatures were detected using the DCMS framework [26], which integrated multiple complementary statistics—Tajima’s D, Nucleotide diversity, iHS and nSL to capture distinct and subtle signatures of selection across the genome. Tajima’s D contrasts the number of segregating sites with the average pairwise nucleotide differences, with deviations from neutrality reflecting selection or demographic events [19]. Nucleotide diversity (π) quantifies the average number of nucleotide differences per site between sequences, with reductions often indicative of selective sweeps [20]. Both metrics were estimated in 50 kb non-overlapping windows using VCFtools v0.1.16 [46]. Haplotype-based methods, including iHS and nSL, detect recent positive selection by analyzing extended haplotype homozygosity. iHS compares haplotype lengths between ancestral and derived alleles [23], whereas nSL evaluates the number of segregating sites within haplotypes, offering enhanced sensitivity to incomplete sweeps [24]. Specifically, iHS is tailored to detect incomplete hard sweeps, while nSL adjusts for recombination variation and is better suited for partial or soft sweeps. By integrating complementary statistics, the DCMS framework generates a robust composite score that enhances detection power and resolution while reducing false positives.

In Pandharpuri buffalo, DCMS identified 1405 putative selective sweep regions containing 1337 candidate genes, several of which are involved in biological pathways relevant to lactation and mammary gland function. Key lactation-related genes, including *ERBB4*, *ESR1*, *SYK*, *INSR*, *PTPN11*, *VAV3*, *MAPK3*, and *PRKG1*, are located within these regions and are known to participate in epithelial proliferation, hormonal signaling, and mammary tissue remodeling. Together, these genomic signals highlight molecular processes plausibly relevant to lactation biology in Pandharpuri buffalo.

*ERBB4* emerged as a key candidate in Pandharpuri buffalo, consistent with its role in regulating mammary epithelial proliferation, differentiation, and ductal development [55,56]. *ERBB4* signaling can induce hyperproliferation in ducts and lobuloalveoli, highlighting that membrane-associated *ERBB4* interactions are critical for regulating epithelial growth [57,58]. In this study, *ERBB4* likely contributes to the ability of Pandharpuri buffalo to sustain lactation under intermittent milking by promoting epithelial survival and tissue remodeling, and preserving milk-secreting capacity. Through its integration with downstream RAS/ERK, PI3K/AKT, and STAT pathways, *ERBB4* may coordinate proliferation, differentiation, and survival signals in response to cycles of milk removal and retention, thereby supporting continuous milk production despite irregular milking intervals. Notably, transcriptomic and functional studies in dairy goats have identified ERBB4 as a hub regulator of mammary gland involution and remodeling via PI3K/AKT signaling, influencing epithelial cell survival, proliferation, and apoptosis during non-lactating stages [59]. Thus selection on *ERBB4* and related network genes may reflect adaptive processes related to mammary gland function in the context of intermittent milking.

*ESR1,* encoding the estrogen receptor α, emerged as a key candidate in Pandharpuri buffalo. In mammary epithelial cells, *ESR1* regulates ductal elongation, epithelial proliferation, and parenchymal expansion during prepubertal and pubertal stages [60,61]. In the context of intermittent milking, *ESR1* signaling may support the maintenance and remodeling of mammary tissue during cycles of milk removal and retention, ensuring sustained epithelial function and milk-secreting capacity. Dynamic coordination of *ESR1* with prolactin and growth hormone pathways, as observed in small ruminants, likely facilitates lactogenic differentiation and casein gene activation [62,63], which could help Pandharpuri buffalo maintain milk production even under irregular milking schedules. Selection on *ESR1* may therefore contribute to the breed’s distinctive ability to balance tissue proliferation, differentiation, and milk yield during intermittent milking.

Among the candidate genes identified, *SYK*, a non-receptor tyrosine kinase, is particularly relevant to intermittent milking due to its role in regulating mammary epithelial proliferation and remodeling. Although traditionally recognized for hematopoietic signaling, *SYK* is also expressed in the bovine mammary gland, where it influences milking cycles and supports milk production. Its elevated expression during the dry period suggests a role in preparing the mammary tissue for subsequent lactation, ensuring epithelial integrity and lactation persistency [64]. Although *SYK* is known for its role in mastitis resistance through modulation of pro- and anti-inflammatory responses in bovine mammary epithelial cells, its signal in this breed may also reflect adaptation to intermittent milking practices, where enhanced immune resilience is advantageous for maintaining udder health under irregular milking intervals [65,66].

The *VAV3* gene has emerged as a versatile candidate in livestock, particularly in mammary gland biology. In cattle, it was identified among genes upregulated in lactating mammary tissue, co-expressed with regulators such as *c-MYC*, *CASP8*, and *LASP1*, suggesting roles in gland growth, remodeling, and secretory function [67]. Among the genes identified, *PTPN11*, a key regulator of the JAK-STAT signaling pathway, plays a central role in lactation biology. In Tharparkar cattle, *PTPN11* appears within selection-signature regions linked to milk yield and milk composition traits [68], and transcriptome-wide analyses in Murrah buffalo and Holstein Friesian confirm its importance in mammary function [69,70]. Functionally, *MAPK3* also enhances milk protein synthesis through its integration with the mTOR signaling pathway [71] and *PRKG1* has been associated with milk yield and fatty acid composition in dairy animals [72,73]. Together, these results uncover the genomic basis and highlight key candidate genes that may govern lactation traits in Pandharpuri buffalo, particularly milk production, lactation efficiency, and the distinctive intermittent milking capacity. The PP interaction analysis further supported these findings by prioritizing genes that consistently overlapped across selection scans and functional enrichment analyses.

Complementing the candidate genes identified in selection scans, the functional enrichment analysis revealed biological processes and pathways directly linked to mammary gland activity and lactational physiology. While a small number of immune-related terms were enriched, these signals are interpreted cautiously, as several implicated genes have well-established pleiotropic roles in mammary remodeling, cellular signaling, and stress adaptation rather than immune-specific selection alone. Key enriched processes included intracellular signal transduction (*VAV3*, *SYK*, *MAPK3*), protein phosphorylation (*MAPK3*, *PRKG1*, *ERBB4*), and calcium ion transport (RYR2), which are essential for epithelial signaling, milk secretion, and mammary remodeling. Enriched pathways such as Wnt signaling (*MAPK3*), cAMP signaling (*VAV3*, *MAPK3*), insulin signaling (*INSR)*, and GnRH signaling (*MAPK3*) regulate hormonal responsiveness, glucose uptake, and milk-component biosynthesis. Notably, insulin- and cAMP-mediated pathways *(INSR*, *MAPK3*, *VAV3*) may support mammary metabolism and maintain milk synthesis during extended inter-milking intervals. Functional pathway–level interpretations are emphasized only where they are supported by concordant DCMS-based selection signals and well-established biological functions of the implicated genes. Consistent with the present findings, DCMS-based genome-wide selection signature studies in livestock have repeatedly identified genomic regions enriched for genes associated with milk production and lactation-related traits across livestock populations” [6,11,15,28,31].

### Future Directions: Validation and Functional Implementation

This study presents the first DCMS-based genome-wide analysis of Pandharpuri buffalo and identifies genomic signals associated with its distinctive intermittent milking phenotype. The within-breed DCMS framework enabled the detection of selection signals specific to this population; however, complementary between-breed or comparative population analyses would further help distinguish breed-specific adaptations from shared or long-term selection effects.

Although the integration of multiple complementary statistics enhances the robustness of DCMS, within-population selection scans remain influenced by demographic history and cannot fully separate selection from demography in the absence of explicit demographic modeling. In addition, as individual-level phenotypic records on intermittent milking were not available for the sequenced animals, the genetic signals are interpreted in the context of established biological functions rather than direct genotype–phenotype associations. Consequently, the identified genomic regions represent candidate selection signals, and further functional validation, including transcriptomic or mammary tissue–specific expression studies, will help clarify their biological relevance. Future studies incorporating larger, independent populations, comparative genomic approaches, and additional layers of genomic variation, including structural variants using long-read sequencing, will strengthen the interpretation of these findings and support genome-informed conservation and genetic improvement of Pandharpuri buffalo.

## 5. Conclusions

This study employed whole-genome resequencing and the DCMS framework to identify within-breed selection signatures in the Pandharpuri buffalo, an indigenous breed characterized by its unique intermittent milking ability. By integrating multiple complementary selection statistics, we identified putative genomic regions harboring candidate genes involved in mammary gland function, hormonal signaling, and milk secretion. Key genes, including *ERBB4*, *ESR1*, *SYK*, *INSR*, *PTPN11*, *VAV3*, *MAPK3,* and *PRKG1*, highlight molecular pathways that may support lactation under irregular milking intervals. Overall, these findings provide a genomic basis for the distinctive adaptation of Pandharpuri buffalo and offer a valuable resource for future functional studies, conservation efforts, and genome-informed breeding strategies.

## Figures and Tables

**Table 1:** Top 20 Genomic Regions with Strongest Selection Signals in Pandharpuri Buffalo.

S.No	Chromosome	Start Position (bp)	End Position (bp)	Window Length (bp)	Number of SNPs	Minimum q-Value	Position of Top SNP (bp)
1	8	93,753,221	93,949,273	196,053	179	1.66 × 10^−9^	93,799,967
2	12	66,038,739	66,099,934	61,196	38	2.16 × 10^−9^	66,099,317
3	20	64,396,901	64,449,434	52,534	36	3.16 × 10^−9^	64,439,325
4	16	8,725,242	8,990,446	265,205	204	6.83 × 10^−9^	8,810,042
5	2	4,741,928	4,799,861	57,934	317	6.98 × 10^−9^	4,778,662
6	15	73,748,472	73,877,721	129,250	446	1.93 × 10^−8^	73,843,490
7	24	16,000,435	16,104,795	104,361	153	3.94 × 10^−8^	16,082,640
8	16	37,625,337	37,699,798	74,462	240	4.73 × 10^−8^	37,668,255
9	5	57,900,501	58,048,083	147,583	149	1.56 × 10^−7^	58,043,435
10	15	75,550,025	75,595,163	45,139	53	2.76 × 10^−7^	75,593,925
11	6	12,778,650	13,081,208	302,559	691	3.10 × 10^−7^	12,794,066
12	18	22,052,257	22,131,818	79,562	195	3.38 × 10^−7^	22,052,587
13	18	21,198,352	21,499,708	301,357	284	7.43 × 10^−7^	21,394,367
14	1	1.34 × 10^8^	1.34 × 10^8^	160,568	76	7.92 × 10^−7^	1.34 × 10^8^
15	14	50,219,895	50,260,964	41,070	63	1.02 × 10^−6^	50,242,895
16	21	16,769,420	16,996,452	227,033	368	1.26 × 10^−6^	16,911,161
17	3	1.38 × 10^8^	1.38 × 10^8^	6834	4	1.29 × 10^−6^	1.38 × 10^8^
18	3	1.31 × 10^8^	1.31 × 10^8^	194,299	286	1.38 × 10^−6^	1.31 × 10^8^
19	12	46,304,985	46,343,138	38,154	4	1.87 × 10^−6^	46,304,985
20	3	29,153,265	29,158,280	5016	6	2.91 × 10^−6^	29,158,239

## Data Availability

The data newly generated in this study are available from the corresponding author upon reasonable request. The publicly available datasets used in this study were obtained from the European Nucleotide Archive (ENA) at the European Bioinformatics Institute under accession number PRJEB39591 (https://www.ebi.ac.uk/ena/browser/view/PRJEB39591) (accessed on 18 September 2024).

## Supplementary Materials

The following supporting information can be downloaded at: https://www.mdpi.com/article/10.3390/cimb48010101/s1.
