# Supplementary material for: Genome-Wide Insights into Intermittent Milking Behavior of Pandharpuri Buffalo
Source: Curr Issues Mol Biol. 2026 Jan 19;48(1):101. doi: 10.3390/cimb48010101 (PMC12839739; doi:10.3390/cimb48010101)
Supplement: Supplementary file 1 [file cimb-48-00101-s001.zip › Supplementary Table S1_Sequencing coverage summary of Pandharpuri buffalo genomes.pdf]

Supplementary Table S1. Sequencing coverage summary of Pandharpuri buffalo genomes

**Sample source**	**Number of animals**	**Coverage (×)**
Public dataset (Dutta *et al.,* 2020)	4	~ 8X
Public dataset (high coverage) (Dutta et al., 2020)	7	~ 28– 41X
Newly sequenced	4	~ 44X
Overall	15	Mean >30×
