# Supplementary material for: Genome-Wide Insights into Intermittent Milking Behavior of Pandharpuri Buffalo
Source: Curr Issues Mol Biol. 2026 Jan 19;48(1):101. doi: 10.3390/cimb48010101 (PMC12839739; doi:10.3390/cimb48010101)
Supplement: Supplementary file 1 [file cimb-48-00101-s001.zip › Supplementary Table S3_Gene Ontology and KEGG Pathway Enrichment Analysis of Genes Under Positive Selection.pdf]

Supplementary Table S3. Gene Ontology and KEGG Pathway Enrichment Analysis of Genes Under Positive Selection

| S.No | Category_Type | Term | Count | PValue |
|---|---|---|---|---|
| 1 | BP | GO:0035556~intracellular signal transduction | 23 | 0.002657885 |
| 2 | BP | GO:0006357~regulation of transcription by RNA polymerase II | 69 | 0.002889285 |
| 3 | BP | GO:0006468~protein phosphorylation | 33 | 0.004792259 |
| 4 | BP | GO:0019731~antibacterial humoral response | 6 | 0.006202982 |
| 5 | BP | GO:0051262~protein tetramerization | 4 | 0.00677994 |
| 6 | BP | GO:0007155~cell adhesion | 12 | 0.010822192 |
| 7 | BP | GO:0000209~protein polyubiquitination | 9 | 0.013127057 |
| 8 | BP | GO:0006338~chromatin remodeling | 8 | 0.013184238 |
| 9 | BP | GO:0070588~calcium ion transmembrane transport | 7 | 0.013802166 |
| 10 | BP | GO:0098609~cell-cell adhesion | 10 | 0.016868595 |
| 11 | BP | GO:0007187~G protein-coupled receptor signaling pathway, coupled to cyclic nucleotide second messenger | 4 | 0.035500337 |
| 12 | BP | GO:0071786~endoplasmic reticulum tubular network organization | 3 | 0.038681824 |
| 13 | BP | GO:0035335~peptidyl-tyrosine dephosphorylation | 3 | 0.038681824 |
| 14 | BP | GO:0050852~T cell receptor signaling pathway | 5 | 0.038886597 |
| 15 | BP | GO:0007165~signal transduction | 23 | 0.039666888 |
| 16 | BP | GO:0000079~regulation of cyclin-dependent protein serine/threonine kinase activity | 5 | 0.043510401 |
| 17 | BP | GO:0008104~protein localization | 4 | 0.048319144 |
| 18 | CC | GO:0005634~nucleus | 166 | 1.67953E-05 |
| 19 | CC | GO:0042734~presynaptic membrane | 6 | 0.004166785 |
| 20 | CC | GO:0005783~endoplasmic reticulum | 26 | 0.005114493 |
| 21 | CC | GO:0031234~extrinsic component of cytoplasmic side of plasma membrane | 5 | 0.016775025 |
| 22 | CC | GO:0042383~sarcolemma | 5 | 0.016775025 |
| 23 | CC | GO:0009986~cell surface | 12 | 0.027743698 |
| 24 | CC | GO:0005666~RNA polymerase III complex | 4 | 0.047276489 |
| 25 | MF | GO:1990782~protein tyrosine kinase binding | 6 | 2.76411E-05 |
| 26 | MF | GO:0004090~carbonyl reductase (NADPH) activity | 7 | 4.25409E-05 |
| 27 | MF | GO:0005515~protein binding | 146 | 0.000160684 |
| 28 | MF | GO:0005509~calcium ion binding | 46 | 0.000781252 |
| 29 | MF | GO:0005524~ATP binding | 78 | 0.00193754 |
| 30 | MF | GO:0004715~non-membrane spanning protein tyrosine kinase activity | 7 | 0.002264122 |
| 31 | MF | GO:0005388~P-type calcium transporter activity | 4 | 0.005219419 |
| 32 | MF | GO:0030414~peptidase inhibitor activity | 6 | 0.011683946 |

| | | | | |
|---|---|---|---|---|
| 33 | MF | GO:0004842~ubiquitin-protein transferase activity | 8 | 0.013211666 |
| 34 | MF | GO:0019901~protein kinase binding | 9 | 0.013720398 |
| 35 | MF | GO:0003682~chromatin binding | 12 | 0.015072085 |
| 36 | MF | GO:0005178~integrin binding | 8 | 0.020982098 |
| 37 | MF | GO:0008046~axon guidance receptor activity | 3 | 0.030632187 |
| 38 | MF | GO:0004725~protein tyrosine phosphatase activity | 7 | 0.035198827 |
| 39 | MF | GO:0004672~protein kinase activity | 12 | 0.039197175 |
| 40 | MF | GO:0005031~tumor necrosis factor receptor activity | 3 | 0.04153191 |
| 41 | Pathway | bbub04310: Wnt signaling pathway | 20 | 0.000442993 |
| 42 | Pathway | bbub05204: Chemical carcinogenesis - DNA adducts | 10 | 0.00336633 |
| 43 | Pathway | bbub00790: Folate biosynthesis | 8 | 0.003940536 |
| 44 | Pathway | bbub00980: Metabolism of xenobiotics by cytochrome P450 | 10 | 0.004557862 |
| 45 | Pathway | bbub04024: cAMP signaling pathway | 22 | 0.005416881 |
| 46 | Pathway | bbub04120: Ubiquitin mediated proteolysis | 16 | 0.005820052 |
| 47 | Pathway | bbub04022: cGMP-PKG signaling pathway | 17 | 0.005850693 |
| 48 | Pathway | bbub04922: Glucagon signaling pathway | 12 | 0.011082097 |
| 49 | Pathway | bbub04911: Insulin secretion | 10 | 0.018504059 |
| 50 | Pathway | bbub04930: Type II diabetes mellitus | 7 | 0.021219537 |
| 51 | Pathway | bbub04520: Adherens junction | 10 | 0.025763956 |
| 52 | Pathway | bbub04961: Endocrine and other factor-regulated calcium reabsorption | 7 | 0.030487916 |
| 53 | Pathway | bbub04934: Cushing syndrome | 14 | 0.0319158 |
| 54 | Pathway | bbub04912: GnRH signaling pathway | 10 | 0.032872054 |
| 55 | Pathway | bbub04261: Adrenergic signaling in cardiomyocytes | 14 | 0.034862431 |
| 56 | Pathway | bbub04925: Aldosterone synthesis and secretion | 10 | 0.041235204 |
| 57 | Pathway | bbub04928: Parathyroid hormone synthesis, secretion and action | 11 | 0.044286958 |
| 58 | Pathway | bbub05412: Arrhythmogenic right ventricular cardiomyopathy | 9 | 0.046952427 |
