# Supplementary material for: Genome-Wide Insights into Intermittent Milking Behavior of Pandharpuri Buffalo
Source: Curr Issues Mol Biol. 2026 Jan 19;48(1):101. doi: 10.3390/cimb48010101 (PMC12839739; doi:10.3390/cimb48010101)
Supplement: Supplementary file 1 [file cimb-48-00101-s001.zip › Supplementary Table S4_Top 15 hub genes identified from the STRING protein–protein interaction network, ranked using the Maximum Clique Centra.pdf]

Supplementary Table S4. Top 15 hub genes identified from the STRING protein–protein interaction network, ranked using the Maximum Clique Centrality (MCC) method

Rank	Ensembl Name	Score	Symbol
1	9913.ENSBTAP00000006216	937	GRB2
2	9913.ENSBTAP00000002653	889	PTPN11
3	9913.ENSBTAP00000015730	511	FYN
3	9913.ENSBTAP00000020771	511	VAV3
5	9913.ENSBTAP00000001995	414	YES1
6	9913.ENSBTAP00000070101	393	SYK
7	9913.ENSBTAP00000071940	346	ERBB4
8	9913.ENSBTAP00000069204	242	TXK
9	9913.ENSBTAP00000074449	224	ESR1
10	9913.ENSBTAP00000023349	184	KITLG
11	9913.ENSBTAP00000034966	153	ITK
12	9913.ENSBTAP00000054333	132	FGF17
13	9913.ENSBTAP00000021507	121	MAPK3
14	9913.ENSBTAP00000015172	120	HSPA9
15	9913.ENSBTAP00000016858	107	INSR
